# Cultivation of *Pseudochlorella pringsheimii* for biodiesel production in a scalable indoor photobioreactor: case studies from Egypt

**DOI:** 10.1186/s43141-022-00450-0

**Published:** 2023-03-02

**Authors:** Hanaa Abd El Baky, Gamal El Baroty

**Affiliations:** 1 Plant Biochemistry Department, National Research Centre, Dokki, Cairo, Egypt; 2 Biochemistry Department, Faculty of Agriculture, Cairo University, Cairo, Egypt

**Keywords:** Biodiesel, *Pseudochlorella pringsheimii*, Fatty acid, Photobioreactor, Nutrient deficiency

## Abstract

**Background:**

Enhancement of lipid accumulation is the major strategy to improve the commercial feasibility of microalgae as a source for biodiesel production. *Pseudochlorella pringsheimii* (Formally was named as *Chlorella ellipsoidea*) green microalgae strain was chosen with respect to their ability as a potential source to produce high lipids content, could be used for the production of biofuel, which can be an alternative renewable energy source instead of fossil fuels.

**Results:**

Initially, the *Pseudochlorella pringsheimii* microalgae was evaluated on the basis of tested at Lab scales 2 L by applicable different nutrient individual of N, P, Fe conditions in BBM medium concentrations for choosing the best concentrations induce lipid contents and productivity to cultivate in large scale in the 2000 L PBR. The suitable concentrations of nutrients with highest lipid contents were obtained under deficient of nitrogen (1.25 gL^−1^, limited N) and phosphorus (0.1 mg L^−1^, limited P) coupled with high iron concentration (10 mg L, rich Fe) and CO_2_ (6%). Therefore, their collective of nutrients was applied to culture of microalgae cells at large scale in 2000 L photobioreactor (PBR model), which, this techniques was used to quantify high lipid contents (25% w/w) and high lipid productivity (74.07 mgL^−1^ day^−1^). The inducted lipid conversion to biodiesel via transestrification process was 91.54 ± 1.43%. The fatty acid methyl esters (FAMEs profile by means of GC/MS resulted in C16:0, C18:1, C18:2, C18:3 as a main constituents. With regard to physical–chemical property (such as density, kinematic viscosity, gravity, and certain number), the *Pseudochlorella pringsheimii* biodiesel have biofuel properties, in accordance with appropriate biodiesel properties, as ASTM and EU standards, that thereby referring to high quality biodiesel.

**Conclusions:**

*Pseudochlorella pringsheimii* cultured in large scale in photobioreactor under stress condition have a high potential of lipids production with high quality of FAMEs that can be used as a promising biodiesel fuel. It has also a potential to be applied for commercialization based on the techno-economic and environmental impacts.

## Highlights

* *Pseudochlorella pringsheimii* showed a high potential of lipids 25% lipid accumulation.

* *Pseudochlorella pringsheimii* could be cultured at large scale in potobioreactor, as potential feedstock for biodiesel production.

*Bodiesel FAME contains C 16:0, C 18:1 and C 18:2 as a maim FAM.

*N, P starvation collective with high Fe^3+^was the main driver behind lipid accumulation.

* *Pseudochlorella pringsheimii* biodiesel have appropriate biodiesel properties, as ASTM and EU standards.

* *C. ellipsoidea* can be used as a potential feedstock for biodiesel production.

## Background

The population of Egypt country is more the 100 million and has the about 5% annual growth rate. It will be about 150 million with respect to population in 2050, as estimated by economic survey of Egypt in 2010 (https://www.populationpyramid.net/egypt/2050/). The fuel poverty has increased, which may be un-expected increase in fuel prices. Therefore, this leads to an increase in global fuel demand and steep pricing. Since, the economics of a county is directly related to the energy availability and its development depends on the energy consumption. Thus, Egypt has a strong potential for bio-energy production, from the local available feedstocks, to meet the increase in energy demand and to find alternative and non-conventional resources of energy. Also, research development (RD), infrastructure, commercialization and biotechnology development are integrating at national level to production biofuel at large scales. The non-edible oil and others feedstock’s can be used for biodiesel production as renewable and sustainable energy resources, that had environmental benefits, it can reduce CO_2_ and others greenhouses gas (GHG) emissions. Current scientific researchers have focused on finding sustainable forms of energy. Thus, the Government has taken a positive step to support finding a renewable energy through Scientific researchers through establishing many finance grants to promote and develop alternative bio-energy, to move in future to use of biodiesel (> 20%).As reported in previous works, some species of microalgae can produce and accumulate lipids contents up to 20–50% of their dry weight when grown under defined conditions, which needs more consideration, commercialization and practical applications [1–3]. For instants, some species of microalgae grow under stress conditions have a high ability to accumulate lipids contents up to 20–50% of their dry weight [4–7]. Thus, it can produces a tremendous quantity of lipids (58,000 L/hr) by several times as much as in soybeans (446 L/ha), Jatropa (1890 L/hr) or Canola (1190 L/hr), when grown on the same area land [8, 9].

However, Egypt has a unique geographical location and suitable climate, so there are many options for the solar and wind energy production, but at the present time the production of wind and solar energy is low. Therefore, it should be promote the technical requirements for commercializing biodiesel from microalgae, was found to be environmentally friendly (characterizes by sulfur-free, nontoxic, less emission of gaseous pollutants and biodegradable) and less production cost [10]. Commercial biodiesel (mono alkyl esters of fatty acids derived from trans-esterification of oil/fats with alcohol) is currently produced mostly from energy crops such as soybean, rapeseed, palm oil or corn. However, the production of biodiesel from these crops is not sustainable, that they not only occupy agriculture land but also many of them are food resources [4]. Microalgae offer many advantage over traditional seed oil crops to produce bio-fuels, have high lipid productivity and photosynthetic efficiencies, higher environmental adaptability, no competition with food or arable land, rapid fixation of environmental carbon gases, cultivation on low water quality and year-round cultivation [11]. Microalgae also, can be grown on marginal soils and the possibility of using waste streams, including carbon dioxide, as a source of nutrients for cell growth and development as compared to terrestrial plants [7]. Moreover, algae can accumulates high quantity of lipids under conditions of nutrient depletion such as sulfur, nitrogen, phosphorus and iron or in physical stresses conditions, e.g. temperature, pH, salinity and high light [2]. Furthermore, it could produce high-value chemicals by-products by biorefinery process (vitamin, pigments such as β-carotenes, astaxanthin, sulfated polysaccharides, PUFAs). The marked application of these chemicals by-products, lead to reduce high cost of microalgae-based biodiesel, to be economically competitive and significantly restricted their industrialization [3, 12].

On other hand, the biodiesel production from algae lipids is more technically sound and cost-effective than traditional seed crop oils and animal fats via transesterification reaction [9]. Algal biodiesel production can cover the ever increasing demand on fossil fuel through the establishment of large-scale photobioreactor on non-arable lands to produce a large amount of algal biomass thereof oil can be used for biodiesel production.

This work, focused primarily on cultured of *Pseudochlorella pringsheimii* at lab scales to optimized nutrient elements (carbon as CO_2_, N, P, Fe ions concentrations), to maximize the lipid accumulation and lipid production. The assessment of the combined effects (as a nutrient stress strategy) of CO_2_, N, P, Fe concentration (as the most important and applicable parameters are combined) to enhance efficiency of lipid accumulation, biomass productivity/growth rate and lipid composition. The microalgae cells *cultured* in PBR at large scales to investigate the suitability of lipid for biodiesel production. The lipid physiochemical properties, profiling of biodiesel fatty acid methyl ester were synthesized by transesterification reaction, and its properties were characterized and compared with specifications set by ASTM and EN standards.

## Methods

### Reagent and chemicals

All the chemicals and solvents were purchased from Sigma Aldrich and E. Merck CO. Other fine chemicals not mentioned here were of analytical grade and obtained from standard sources. The Millipore Milli Q plus system (Mark, Darmstadt, Germany) was used to prepare the high purely water.

#### Microalgae strain and cultivation

##### Microalga strain and pre-culture condition

*Pseudochlorella pringsheimii* strain was preserved in a Bold´s Basal Medium (BBM, pH 6.6) medium under optimum conditions, as reported in a previous work. The pure strain isolated from water resources had been deposited in Egyptian culture collation (accession number EMCCN 3044), National Research Centre (Giza, Egypt).

##### Pre-culture condition

For optimize the nutrient grown, employing different concentrations of (CO_2_ and bicarbonate) as a carbon sources, bicarbonate at 2, 4 and 8 g L^1^ and CO_2_ enriched air supply 4, 8, 16% in two liter Erlenmeyer flasks each containing 1.80 L of BBM medium, was achieved, that to ensure the better results for accumulation high lipid contents. The cultures was grown photo-autotrophically under 10 white florescent light lamps (Philips 40 W) provided an illumination of 2500 lx. After the first steady-state condition was reached, CO_2_ at 8% as a carbon source was selected due to given higher biomass dry weight and biomass productivity. Further treatments, by study the effect of the three elements availability (N, P, S) in BBM growth medium (at Lab scales) was determined to optimize and develop the economic viability of *Pseudochlorella pringsheimii* as feedstock biodiesel. Therefore, the algae was growing in different medium containing serial concentrations of nitrogen (KNO_3_, 0.0, 0.125, 0.25, 0.50 g/L), phosphorus (KH_2_PO_4_, 0.0, 0.13, 0.175, 0.14 g/L), iron (FeSO_4_, 0.0, 5.0, 10.0 mg/L) to enhance lipid yield and high lipid productivity. In all cultivated flasks, conductivity, salinity, pH and temperature were measured every two days with Hanna (HI 09,812–5, HANNA instruments, USA) conductivity meter. The purity of cultures was periodically checked by microscopic observation following taxonomy guidelines.

### Cultivation of *Pseudochlorella pringsheimii* in 2000 L photobioreactor

A algae was cultivated in BBM containing a combination of limited two element ( 0.125 g/L Nitrogen and 0.13 g/L phosphorus) and optimal levels of CO_2_ and 10 mg/L Fe as the most suitable nutrient levels, in 2000 L (large scale) tubular photobioreactor for enhance lipid accumulation for biodiesel production. The PBR was continuously aerated by compressed-air from an air pump through the static sparger and air flow rate was controlled by a flow meter. The cultivation system was maintained at 24-h photo-period via twenty cool-white fluorescent lights (Philips, TL-D 36 W/54–765) that was illuminated with an intensity of 130–170 µmol m^−2^ s^−1^_._

### Harvesting

At the end of each batch run (the experiment was repeated 3 times and average), cultures were collected, filtered, washed with distilled water to remove any residual soluble salts, centrifuged at 3000 xg for 15 min at 4˚C and the pellets was frozen until using.

### Growth measurement

The growth of *Pseudochlorella pringsheimii* was assessed every two days, during the 16 days of the cultivation period (log and stationary phase), using the dry cell weight method and optical density of the culture suspension. The optical density (OD) was read at 680 nm, and the biomass density of the culture suspension was calculated. A standard curve was established by correlation of the absorbance values (OD_680_ nm) and dry cell weight (g/L) as follows: Dry weight g/L = 1.074 X OD_680_ + 0.09855.

### Determination of dry cell weight

To determine the dry cell weight, a known volume of (10 ml) culture suspension was centrifuged at 6000 xg for 15 min. The collected cells pellet was several times washed with distilled water to get rid of medium of any traces nutrients. Then, the biomass pellet was dried in an oven at 60 °C to get the constant weight and their dry weights were determined and kept for further analysis.

Microalgae growth parameters.

During the cultivation period, growth parameters were determined as follows:

#### Biomass productivity (PB)

The dry biomass produced (g L^−1^day^−1^), during the stationary growth phase was determined. A known volume of the algal suspensions were centrifuged (6000 xg, 15 min) and the resulting pellets were washed with deionized water, dried at 70°C for 48 h and their dry weights were determined gravimetrically.

Specific growth rate (SGR, µg/ day)

The specific growth rate (SGR µg/ day) of culture was determined using the following equation: SGR (µg/ day) = ln (X_1_- X_2_)/ t_2_ – t_1_: Where, X_1_ = Biomass concentration at the end of selected time interval, X_2_ = biomass concentration at the beginning of selected time interval, and t_2_ –t_1_ = elapsed time between selected time in the day.

### Determination of total chlorophyll a (Chl a)

A known volume (10 ml) from the each culture was harvested by centrifuging, and then algal pellet was washed several times with distilled water and re-suspended in 10 ml of 95 % methanol and then extracted aided by ultrasonic processor and centrifuged. The supernatant containing the pigment was transferred to a volumetric flask (10 ml) and volume was made up to 10 ml by adding pure methanol. A blank with 100% methanol was run simultaneously. The Chl *a* content in the biomass was spectrophotemerically (UV-VIS, Thermo Scientific, USA) at 648.6 nm and 664.2 nm [13], using the follows Eq. Chl a = (13.36 A _664_− 5.19 A _648_) x 8.1/dry weight (mg/ g dw).

### Determination of total lipids contents

To determine the total lipid content in dried microalgae cells, 2 g of biomass was extracted with 30 ml hexane: methanol (v/v, 1:1) and then 3 ml of distilled water was added. The contents were mixed for 10 min in a shaker, and then left at room temperature (25 °C) for 10 min. The organic layer was separated by a 50 ml separating funnel, several times washed with 5% NaCl solution. Then, the organic extract was evaporated under vacuum to smallest volume, and evaporated under nitrogen stream. The lipid yield was calculated in percentage gravimetrically (%).

### Lipid productivity (LP)

Lipid productivity (LP, mgL^−1^ day^−1^) was calculated by the following equation LP = ¼ PB – LC (where P is the sample dry weight at the end of the logarithmic growth phase and LC is the total content).

Determination of physical − chemical properties of *Pseudochlorella pringsheimii* lipid.

Based on AOCS Method [14], acid value (AV, Ca5a-40), saponification value (cd 3c-91), iodine value (IV, Cd1-25) and peroxide value (PV Cd8-53) of microalgae lipids were determined. The molecular weight of the oil (M) was calculated from saponification and acid value according to M = 168,300/ SV − AV formula. The viscosity was measured with capillary viscometer in a constant temperature in water bath at 40 °C.

### Biodiesel preparation

BIodIESEL from algal lipids was derived by acidic trans-esterification method. Algae lipid was mixed with methanol with 1:56 ratio (weight ratio) and the reaction was carried out at 45 °C for 4 h in the presence of sulfuric acid (H_2_SO_4_) as catalyst with1:1 weight ratio of catalyst to lipids. The upper biodiesel layer was separated by a separating funnel, washed several times with 5% NaCl solution to remove any traces of methanol and glycerol. Then, the biodiesel was dried over anhydrous sodium sulfate and collected and evaporated at 45 °C to constant weight. The biodiesel yield was determined gravimetrically by the following equation, Biodiesel yields (%) = [biodiesel mass (g) / algae mass (g) X lipid content %] × 100%

### Biodiesel fatty acid methyl ester profile

The fatty acid methyl ester (FAME) composition of algal biodiesel was analyzed by using GC–MS (HP-6890 gas chromatograph connected to an HP 5973 mass selective detector) at 70 eV (m/z 50–550 amu; source at 230 °C and quadruple at 150 °C) in the electron impact mode with a TR-FAME-ms capillary column (30 m × 0.25 mm i.d., 0.25 μm film thickness, 75% cyanophenyl-silxane, Thermo Co, USA). The oven temperature was programmed for 2 min at 80 °C and raised to 280 °C at 4 °C/min and maintained for 5 min at 280 °C. Helium was used as carrier gas (flow rate of 1.2 ml/min). The inlet temperature was maintained at 300 °C. Structural assignments were based on interpretation of mass spectrometry fragmentation (NIST 11 MS spectra) and confirmed by comparison of retention times as well as fragmentation patterns of authentic standard FAMEs Mix (Supelco 37 component FAME mix, purity > 98.0%).

### Characterization of properties of biodiesel

The direct measurement of critical biodiesel quality was based on FAMEs composition makes the estimation of fuel property easy and quick, which is important in biodiesel-based application [15, 16]. Thus, the biodiesel property of algae, the degree of unsaturation (DU), kinematic viscosity (KS), specific gravity (SG), cloud point (CP), iodine value (IV), a certain number (CN) and higher heating value (HHV) long-chain saturated factor (LCSF), and cold filter plugging point (CFPP), were determined by empirical equations from FA composition [15–18]. The iodine value acid value, peroxide value and saponification value (Cd 3c-91) were determined as reported in AOCS [14]. All determinations for biodiesel properties were conducted at least three times for each sample, and the results were averaged.

### Statistical analysis

All experiments were achieved in triplicates and results were reported as means of three replications and standard deviation (SD). The experimental results were analyzed by one-way ANOVA (at *P* value < 0.05) using the statistical software package “COSTAT” a product of Cohort Software Inc., Berkeley, California, USA.

## Results

### Effect of carbon on biomass growth and lipid yield

**Fig. 1:**
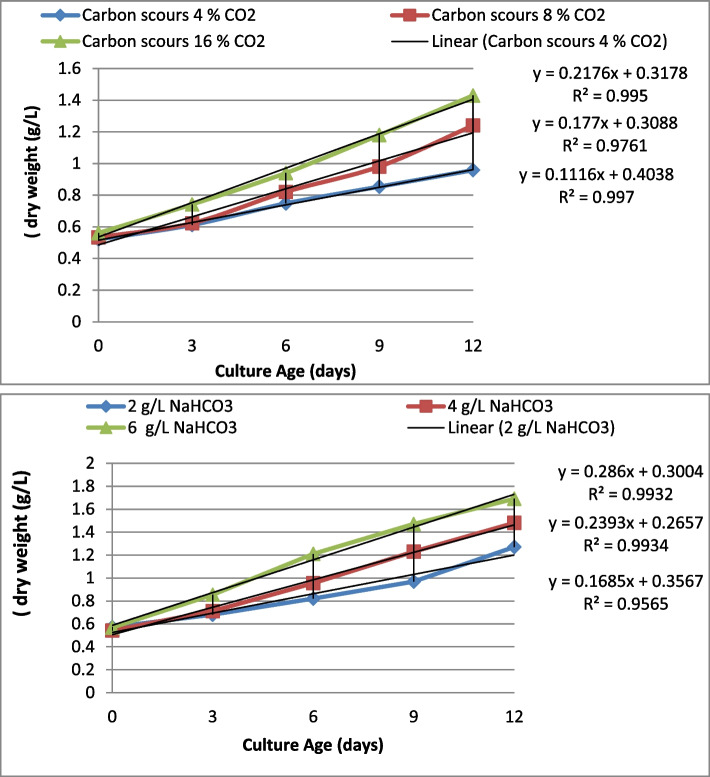
Impact of carbon source and concentrations on *Pseudochlorella pringsheimii* growth and and oil yield

To optimization the growth condition for quantified of lipid content and lipid productivity for biodiesel production of *Pseudochlorella pringsheimii,* microalgae is cultured in different nutrient conditions (Carbon sources and concentration, N, P and N) continuously at indoor-laboratory scales (2 L flasks). Table 1 and Fig. 1 are presented the growth data of microalgae cultured in BBM medium supplemented with CO_2_ as a carbon source, three concentrations (CO_2_ 4%, 8%, 16%) was used (as a limited L, optimal O, rich R, concentrations) over 12 day of incubation. The data revealed that significant trend on biomass yields (BM, based on dry weigh dw) and total lipid contents (TL) were observed (*P* > 0.5%) among tested cultures. At these concentration the biomass of 1.00, 1.24 and 1.34 g/L, respectively and lipid yield of 4.6%, 18.58% and 21.65%, respectively was obtained after 12 of incubation. At 16% of CO_2_ gases, there was significant increase in lipid yield that obtained in 8% CO_2_ culture, but no significant effect was recorded.

**Table 1:** Impact of carbon scours and concentrations on *Pseudochlorella pringsheimii* growth ( dry weight g/L) and oil contents (%)

Carbon scours & Concentrations	Culture Age (days)					Oil content %
**CO**_**2**_	**0**	**3**	**6**	**9**	**12**	
4% CO_2_	0.521 ± 0.04	0.612 ± 0.06	0.748 ± 0.07	0.854 ± 0.06	0.958 ± 0.05	4.65^a^
8% CO_2_	0.534 ± 0.06	0.623 ± 0.05	0.821 ± 0.05	0.981 ± 0.08	1.24 ± 0.06	18.58^b^
16% CO_2_	0.561 ± 0.05	0.742 ± 0.06	0.94 ± 0.06	1.18 ± 0.07	1.43 ± 0.07	21.65^c^
NaHCO_3_	**0**	**3**	**6**	**9**	**12**	
2.0 g /L	0.571 ± 0.03	0.681 ± 0.03	0.821 ± 0.05	0.968 ± 0.06	1.27 ± 0.05	5.36^a^
4.0 g /L	0.543 ± 0.06	0.711 ± 0.04	0.954 ± 0.06	1.23 ± 0.07	1.48 ± 0.06	15.68^b^
6.0 g /L	0.568 ± 0.03	0.854 ± 0.06	1.21 ± 0.07	1.47 ± 0.05	1.69 ± 0.07	19.25^c^

Data represent means ± SD values from three independent experiments. The Values of the oil contents in column with the different superscript are significantly different (LSD = 0.61 at level *P* < 0.05)

The different letters in the same column are significant differences between the treated samples

Experimental set of sodium bicarbonate salt of NaHCO_2_, as a carbon salt, was cultured in BBM medium supplemented with three concentrations of 2.5, 5.0 and 0.5 mmol L^−1^ for 12 days. As shown in Table 1 and Fig. 1, the biomass yields (dw) and total lipid contents (TL, in parentheses) were 0.122 mg/L (5.36%), 0.191 mg/L (5.85%) and 0.201 mg/L (19.25%), respectively. These values were lower than get in CO_2_ cultures.

### Impact of N level on biomasses and lipid content

**Fig. 2:**
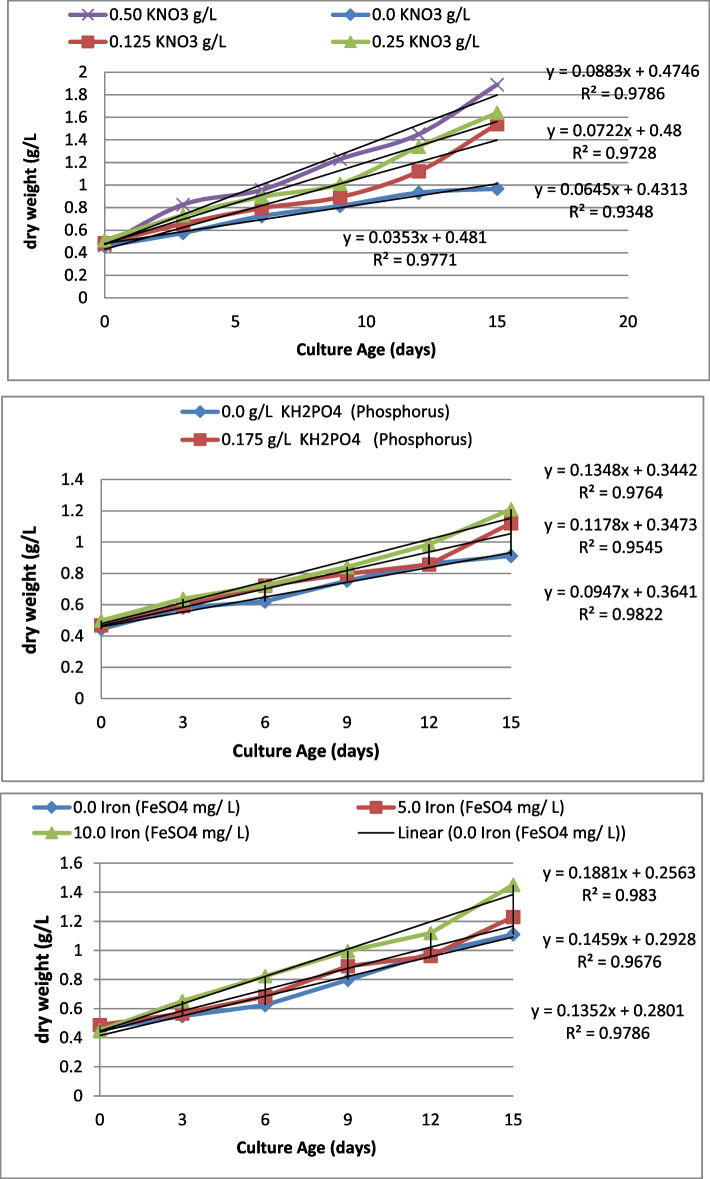
Impact of elements concentrations on *Pseudochlorella pringsheimii* Biomass ( dry weight (g/L)

Figure 2 and Table 2 show the time course biomass profile of *Pseudochlorella pringsheimii* grown in MMB medium supplemented with different concentrations of N (nitrogen source KNO_3_. Based on the original nitrogen source concentration, the optimum level was 0.25 g /L, different sets of nitrogen concentrations: free N (0 N free), ½ (0.125 g/L), optimum (0.25 g /L) and double (0.50 g/L) of optimum was used to investigate the effect of nitrogen on growth and lipid content of the microalgae cells. As shown in Fig. 2 the algal growth was increased after a short lag phase of 3 days followed by a logarithmic phase and continuous the log phase till 15 days. As shown in Fig. 2, by increasing the N concentration the cells growth was increased. In the absence of N free source (0 g/ L free N) little and slowly growth was observed and the cells appeared bleached. In contrast, in rich N sources (double the N concentration 0.50 g/L, rich N), at 15 days of incubation, the maximum biomass (dw) concentration of 1.86 g/L was recorded. Dry weight of 1.64, 1.54 and 0.96 g/L was observed in cultures with 0.25 g L (optimum), 0.125 (1/2 of optimum, Limited) and 0.0 KNO3 (free N), respectively (Fig. 2). An increasing trend is observed in lipid content as the concentration of N was decreased. Also, at the zero nitrogen free and 0.125 N (1/2 of optimum N, limited) concentrations, both cultures showed higher lipid accumulation in comparison to that of 0.25 N (optimum) and 0.50 g/L doubled N (rich N) cultures. When the concentration of N was doubled, the lipid content was decreased to be 4.32% dry cell weight as opposed to optimum N (8.14%). Thus, concentration of nitrogen source was increased the lipid contents were decreasing as a function for N levels. For example, as a concentration of nitrogen source was decreased from 0.25 g L N (1/2 of optimum) to 0.125 g L (1/2 of optimum), the lipid content in cells increased to be 3.3 folds. The *Pseudochlorella pringsheimii* cultures did not show much difference (28.14%) in lipid content when grown in 0.125 g L N than that in zero nitrogen culture. The correlation coefficient between the N concentrations and the amount of biomass was ranged from 0.934 to 0.978, which indicates a strong positive linear relationship.

**Table 2:** Impact of elements concentrations on *Pseudochlorella pringsheimii* Biomass ( dry weight (g/L) and oil contents

Elements Concentrations	Culture Age (days)						Oil contents %
	**0**	**3**	**6**	**9**	**12**	**15**	
**Nitrogen (KNO**_**3**_** g/L)**							
0.0	0.458 ± 0.03	0.576 ± 0.05	0.725 ± 0.04	0.815 ± 0.04	0.932 ± 0.08	0.967 ± 0.04	**29.51 **^**a**^
0.125	0.483 ± 0.0	0.658 ± 0.04	0.795 ± 0.06	0.893 ± 0.06	1.12 ± 0.07	1.54 ± 0.06	**28.14 **^**b**^
0.255	0.511 ± 0.04	0.733 ± 0.05	0.895 ± 0.07	1.01 ± 0.07	1.34 ± 0.06	1.64 ± 0.08	**8.14 **^**c**^
0.50	0.465 ± 0.04	0.827 ± 0.06	1.681 ± 0.04	2.65 ± 0.08	2.98 ± 0.09	4.48 ± 0.2	**4.32 **^**d**^
**Phosphorus ( KH**_**2**_**PO**_**4**_** g/L)**							
0.0	0.444 ± 0.04	0.581 ± 0.05	0.621 ± 0.04	0.754 ± 0.04	0.862 ± 0.04	0.912 ± 0.09	**15.65 **^**a**^
0.175	0.469 ± 0.05	0.593 ± 0.04	0.721 ± 0.05	0.798 ± 0.04	0.857 ± 0.08	1.12 ± 0.08	**12.36 **^**b**^
0.35	0.536 ± 0.04	0.589 ± 0.06	0.936 ± 0.06	1.153 ± 0.07	1.223 ± 0.07	1.356 ± 0.07	**4.38 **^**c**^
**Iron (FeSO**_**4**_** mg/ L)**							
0.0	0.458 ± 0.06	0.548 ± 0.05	0.625 ± 0.04	0.798 ± 0.04	0.981 ± 0.04	1.11 ± 0.09	**5.36 **^**a**^
5.0	0.487 ± 0.05	0.567 ± 0.06	0.684 ± 0.06	0.891 ± 0.04	0.962 ± 0.04	1.23 ± 0.08	**7.25 **^**b**^
10.0	0.449 ± 0.06	0.651 ± 0.06	0.823 ± 0.05	0.995 ± 0.06	1.12 ± 0.05	1.45 ± 0.06	**10.25 **^**c**^

Data represent means ±SD values from three independent experiments. The Values of the all parameters [Dry weight (g/L), Chll a (mg/L), Oil content (%) and Oil Yield (g/L)] in the column are significantly different at < 0.05

The different letters in the same column are significant differences between the treated samples

### Impact of P levels on biomasses and lipid content

Different sets of phosphorus P concentrations: free P (P free), (0.175 g/L), and double (0.350 g/L) was added to BBM optimum medium, to investigate the effect of P on growth and lipid content of the *Pseudochlorella pringsheimii.* Growth of algae increased after a high lag phase of 6 days followed by a logarithmic phase continuously till 15 days. As extracted from the Fig. 2, by increasing the P concentration the cells growth was slightly increased. In the absence of P free source (0 g/ L free P) very slowly growth was observed. In contrast, in rich P sources (double the P concentration0.350 g/L, rich P), at 15 days of incubation, the maximum biomass (dw) concentration of 1.356 g/L was recorded. Dry weight of 0.96 g/L and 1.12 g/L was obtained in cultures with free P and 0.175 g L, respectively (Fig. 2). The cultured in P-rich medium produced the lowest lipid contents of 4.38%, followed by the values of 12.36% in P standard medium. Whereas, in Free P BBM medium, the obtained average of lipid content was 15.65%. Thus, statistically there is higher significant difference between the lipid contents of P-rich or P- stander cultures and in the P-deficient BBM medium (*p* > 0.05). In contrast, cultures in rich P medium and standard P BBM BG11 medium had highest biomass concentrations that in free P culture.

### Impact of Fe levels on biomasses and lipid content

In a different sets of Fe concentrations: free Fe (Fe free), optimum (5.0 mg/L), and double (10.0 mg/L) of optimum BBM medium, the growth of the *Pseudochlorella pringsheimii* was increased after a short lag phase of 3 days followed by a logarithmic phase and continuous the log phase until 15 days. The biomass (dw) concentration of 1.11, 1.23 and 1.54 g/L and total lipid of 5.36, 7.25 and 19.25 were obtained in free, 5 and 10.0 mg/L Fe cultures, respectively. The total lipid content in cultures supplemented with high Fe^3+^ concentration exhibited a higher (about 2-folds) than that in free Fe^3+^ iron concentration.

### Impact of combined effect of N, P and Fe ions on growth and lipid content lipid content of the microalge cultured in 2000 L photobioreactor

**Fig. 3:**
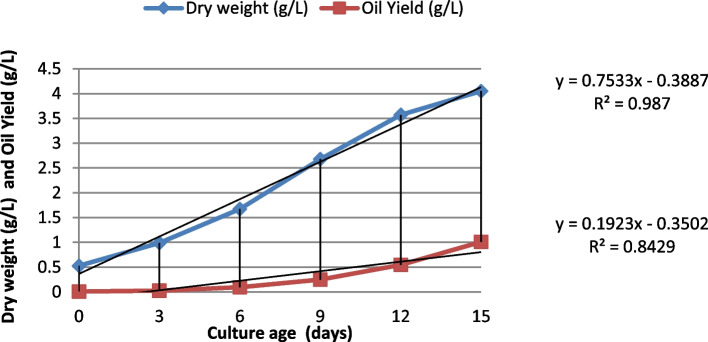
Dry weight and Oil yield of *Pseudochlorella pringsheimii* grown in photobioreactor (2000 L) undeer nitrogen, and Phosphours deficient and high concentration of Iron and corbon dioxide

The individual experimental showed that relative higher lipid content was obtained in the *Pseudochlorella pringsheimii* cultured under: N-, P- starvation and rich Fe ions condition. It should be noted that the N starvation was the most effective strategy to increase lipid content of the microalgae to use as biodiesel feedstocks. Therefore, the algae cells was cultivated at large scales in photobioreactor (PBR, 2000 L) in medium contained a collective nutrients (deficient N and P, rich -Fe^3+^: (N 1.25 and aerated with carbon dioxide gases (8% CO_2_) as a carbon source. Table 3 and Fig. 3, showed higher total lipid and lipid productivity of the *Pseudochlorella pringsheimii* cultured in PBR. Under this condition, the values of biomasses dry weight (4.05 g/L), lipid content (24.98%), lipid productivity (1.01 g/L) and total chlorophyll Chl a (96.31 mg/L) was calculated at late log phase (15 days). However, all growth parameters of algae grow in PBR 2000 L medium had significant increased as factor of cultivation period was increased includes the rate of dry weight accumulation, biomass productivity, and the chlorophyll content (Fig. 3 and Table 3). The experimental was design for production of higher lipid content for biodiesel production at indusial scales. Under this condition, the values of biomasses dry weight (4.05 g/L), lipid content (24.98%), lipid productivity (1.01 g/L) and total Chl a (96.31 mg/L) was calculated at late log phase (15 days). Also, the specific growth rate μ (d^−1^), biomasses productivity (g^−1^L^−1^d^−1^) were 0.274 and 0.270, respectively. The correlation coefficient between the biomasses yield and oil content values (R^2^ was 0.842 to 0.987), which indicates a strong positive linear relationship. This result confirmed the fact that nitrogen and P starvation is the important nutrient for enhance of accumulation of lipid in microalgae.

**Table 3:** Dry weight,Chll a, Oil content and Oil yield of *Pseudochlorella pringsheimii grown in photobioreactor* (2000 L) under nitrogen, and Phosphours deficient and high concentration of Iron and corbon dioxide

Culture Age (days)	Dry weight (g/L)	Chll a (mg/L)	Oil content (%)	Oil Yield (g/L)
0	0.529 ± 0.04	**6.67 ± 0.11**	1.69 ± 0.12	0.019 ± 0.01
3	0.987 ± 0.05	12.32 ± 0.45	2.64 ± 0.13	0.026 ± 0.01
6	1.67 ± 0.06	22.36 ± 0.89	5.64 ± 0.25	0.093 ± 0.01
9	2.68 ± 0.08	41.32 ± 1.98	9.37 ± 0.29	0.251 ± 0.02
12	3.57 ± 0.189	64.32 ± 0.87	15.36 ± 0.42	0.5482 ± 0.03
15	4.05 ± 0.152	96.31 ± 3.9	24.98 ± 1.2	1.01 ± 0.04

Biodiesel production from Pseudochlorella *pringsheimii.*

Microalgae lipids are known to have high free fatty acid content thus acid catalysis become the preferred choice over alkali catalysts for biodiesel conversion. The direct transesterification TE reaction condition was optimized as a way to improve the biodiesel yield of algae lipids. The microalgae biomass cultured at large scales in limited combinations of (N + Fe + P) medium was subjected to a directly TE in one step. The FAME conversion percentage being high biodiesel yield (BY %) and biodiesel productivity was found to be about 97.64% and 0.067 mg^−1^L^−1^d^−1^, respectively (Table 4).

**Table 4:** Summary of *Pseudochlorella pringsheimii* cells cultivation under high biomass production and high lipids production conditions

**Parameters**	Algal Cells grown under high biomass production conditions	Algal Cells grown under high lipids production conditions
Biomass productivity (g^−1^ L ^−1^ d^−1^)	0.289	0.27
Specific growth rate, μ (d^−1^)	0.341	0.274
R2 = coefficient	0.9786	R^2^ = 0.9527
Max. lipids contents (w/w %)	4.32	24.98
Max. lipids productivity(g^−1^ L ^−1^ d^−1^)	0.012	0.067
R^2 =^ coefficient	0.978	0.8429
Max. biodiesel contents (w/w %)	75.33	97.64
Max. biodiesel productivity(g^−1^ L ^−1^ d^−1^)	0.011	0.06
Biodiesel convenrsion yeild %	91.54 ± 1.43	91.54 ± 1.43

### Identification of fatty acid methyl esters (biodiesel)

The profile and characteristic of fatty acids methyl esters (biodiesel, FAMEs, Tables 5 and 6) of *Pseudochlorella pringsheimii* biodiesel prepared with transessterification reaction were comprehensively identified by GC/MS. The major fatty acids methyl esters (> 10%) were found to be 16:0 (6.67%), 16:1 (9c, 12.21) and18:1 (9c, 38.57). The C16:0 (10 5.11%), C 18:2 (c9, c12, 6.47%), 18:3 (c9, c12, c15, 3.96%) and C18:3 (c6, c9, c12, 4.55%) was identified as polyunsaturated fatty acids methyl esters. The following saturated FAMEs were identified, C14:0 (2.33%), C15:0 (5.52%) and C18:0 (4.35%).Complete separation for the long-chain saturated and unsaturated fatty acids FAMEs of C20 and C22 was not achieved, that was present in trace levels and estimated as the sum of the many components, not excess more than (< 0.1%). However, the algal lipid content and fatty acid profile have a high influence on fuel property.

**Table 5:** Fatty acids composition of Pseudochlorella pringsheimii *biodieselat 15 days of cultivation*

**Fatty acids**^**a**^	**Relative content (%)**^**b**^
C_14:0_	2.33
C_15:0_	5.52
C_16:0_	16.67
C_16:1 9c_	12.21
C_16:2 7,10c_	7.24
C_17:0_	5.11
C_18:0_	4.35
C_18:1 (9c)_	38.57
C_18:2 (9,12 c)_	6.47
γC_18:3 (6,9,12 c)_	4.55
C_18:3 α (9,12,15 c)_	3.69

^a^Fatty acid was identified based on the retention time of standard fatty acids and its MS spectram data

^b^The percentage of the fatty acid was evaluated through the peak area

**Table 6:** Evaluation criteria o*f Pseudochlorella pringsheimii* biodiesel

Lipid criteria	Paramerters
Total saturated fatty acids %	33.98
Total monounsaturated fatty acid %	50.78
Total polyunsaturated fatty acids %	21.95
Total unsaturated fatty acids %	72.73
TU/TS ratio	2.14
DU degree	1.02
RO value	4.21

TU/TS ratio: Total unsaturated / Total saturated

DU: Degree of Unsaturated: TMSFA/100 + 2 [Tdi = FA/100] + 3 [T Tri = FA /100] + 4 [Tetra = FA /100]

RO: Rate of oxidation = [%UFA 1 =  × 1 /100] + [%UFA 2 =  × 12 /100] + [%UFA 3 =  × 25 /100] + [%UFA 3 =  × 50 /100] ( =): Number of double ( =): Number of double bonds

The properties of biodiesel *Pseudochlorella pringsheimii.*

The properties of biodiesel of algae are represented in Table 7. The iodine value (IV 89.84 ± 3.33 (g I_2_/100 g)), acid value (AV 0.41 ± 0.09 mg KOH ^−1^ g), saponification value (SV 199.33 ± 1.66 mg of KOH/g) and peroxide value (PV < 0.1 O_2_ meq/kg) was determined based on ACOS methods [14]. The cetan number CN of FAME of microalgae *was* calculated using its saponification value and iodine value. The value of CN was 53.46. The CN is a prime indicator of biodiesel quality and related to the ignition delay time (is the time that passes between injection of fuel into the cylinder and the onset of ignition) and combustion quality. However, the low values of auto oxidative (OS, 0.169) and degree unsaturation (0.97) of microalgae biodiesel was recorded. The density (D at 15 °C, kgm^3^) and viscosity (KV 40 °C, mm^2^/s) of biodiesel were 0.922 ± 0.080 and 4.98 ± 0.24, respectively. Other biodiesel parameters such as the heating value (39.22), LCSF (31.42) and CFPP (82.25) were calculated, which are used to evaluate biodiesel quality in terms of ignition readiness; combustion performance and fuel-line plugging temperature.

**Table 7:** Physiochemical properties of *Pseudochlorella pringsheimii* biodiesel, diesel fuel and biodiesel standard

**Property**	**Units**	**Biodiesel of microalgae**	**Diesel**^**a**^** Fuel**	**ASTM**^**a**^** Biodiesel Standard**
Density	(Kg^−1^L)		0.838	0.86—0.9
Viscosity	mm^2^ S^−1^, at 40 ºC	4.98 ± o.24	1.9—4.1	3.5—5.0
Acid value (AV)	mg KOH ^−1^ g	0.41 ± 0.09	Max 0.5	Max 0.5
Peroxide number	meq/kg	ND	ND	
saponification value (SV)	(mg of KOH/g)	199.33 ± 1.66	….	………
Iodine value	g I_2_/100 g	89.84 ± 3.33	120	Max 120
Cetane number (CN)		53.46	45.9	≥ 47
Oxidiz ability (OX)		0.169	……	> 0.7
DU		97.29	……	……
Heating value	(MJ/kg) HV	39.22		
HHV		41.75		
Flash point (FP)	(°C)	79.5		93
FP		45.87		
LCSF		31.42		….
CFPP		82.25		……

^a^The data about diesel and ASTM biodiesel standard were taken from published literature

DU = MUFA + (2X PUSFA)

CN = (46.3 + [5458/SV]) -(0.225 X IV)

Oxidize ability (OX) = [0.02(%Oleic) + % linoleic + 2 (% linolenic)] / 100

Higher Heating Value (HHv) = 0.4625 V (viscosity) + 39.450

Cloud point (CP), = (HHV -32.12) /0.21

Long-chain saturated factor (LCSF) = (0.1 X C_16_) + (0.5 XC_18_) + (1 XC_20_) + (1.5 X C_22_) + (2 X C_24_)

Long-chain saturated factor (LCSF) = (0.1 X C_16_) + (0.5 XC_18_) + (1 XC_20_) + (1.5 X C_22_) + (2 X C_24_)

## Discussion

Nutrient composition have an important role in metabolic pathway of microalgae constituent includes primary and second metabolites as sources for biofuel, fertilizer, cosmetics, nutraceuticals and pharmaceutical material [2, 3]. Cultivation of macro or micro-algae in depletion and excessive sources of nutrient might affect the quality of biomass and quality and quantity of bio-molecule such as lipid, protein, pigments and polysaccharides. However, investigation on the impact of nitrogen, phosphorus and ferric, which are crucial for the growth of algae has been addressed [1–5]. On other hand, enhancing nutrient utilization efficiently for cultivated of microalgae at large scale cultivation. Hence, this study aims to highlight the concentration of N, P and Fe required for *Pseudochlorella pringsheimii* microalgae cultivation to increasing biomass productivity and lipid contents and to produce fatty acid quality for biodiesel production.

In this study, *Pseudochlorella pringsheimii* was grow firstly at lab scales (2 L flasks) in order to optimizes the suitable nutrient condition, to get a high growth rate, high lipid content for biodiesel production. The carbon concentrations, and three elements availability (N, P, Fe) in the microalgae growth medium required for the metabolic pathway switch to lipid accumulation was determined.. The data microalgae revealed that the lipid content and lipid productivity of microalgae was significantly affects by treated with carbon source and concentration (*p* < 0.05), the higher values was obtained in 8% and 16% gaseous CO_2_ cultures (Table 1). The correlation coefficient (R^2^) between the microalgae biomass and the carbon sources and level (Fig. 1) in nutrient medium throughout the cultivation period was found to be very high (R^2^ ranged from 0.956 – 0.997). However, lipid content lipid productivity of *Pseudochlorella pringsheimii* cultures in sodium bicarbonate salt (NaHCO_2_), the biomass yields (as dw) were found to be lower than in CO_2_ cultures, that suggests the occurrence of NaHCO_2_ stress conditions induced by high bicarbonate levels in the medium due to excess osmotic pressure [4]. The CO_2_ gases could be more economically sustainable than that supplied by an exogenous organic carbon source [19]. The high lipid productivity and lipid contents are considered as the most important desirable characteristic of chosen the microalgae strains and nutrient for biodiesel production [20]. However, the high concentration of CO_2_ gases had a significant effect on the growth of microalgae that the utilized CO_2_ in culture will be converted to carbonic acid (H_2_CO_3_), as result reducing the pH value of the culture [4]. Therefore, to obtain enhanced biomass and lipid production requires optimal CO_2_concentration. In this regard, the *Dunaliella salina* had high lipid content when cultivated at 6% to 10% CO_2_ gases as compared with that cultivated in 1% CO_2_. However, 8% CO_2_ was used as carbon source for cultivation of several microalgae species to provide the best result in terms of higher oil yield and lipid productivity [4, 5 and 21].

Nitrogen is an essential macronutrient for microalgae growth and plays an important role in primary constituents like protein, lipid and nucleic acids, chlorophyll and in syntheses the energy transfer molecules viz adenosine triphosphate ATP [2, 5]. As showed in Fig. 2 and Table 2, *Pseudochlorella pringsheimii* grow in MMB medium with different concentrations of N (KNO_3_, free N (0 N free), ½ (0.125 g/L), and double (0.50 g/L) of optimum) had a significant differences in biomass and lipid content. AsN concentration increasing the biomasses and cells growth was increased with high correlation coefficient (R^2^ ranged from 0.978 to 0. 934). In the absence of N free source (0 g/ L free N) little and slowly growth was observed and the cells appeared in bleached form. In contrast, in rich N medium (double N concentration 0.50 g/L, rich N), the maximum biomass of 1.86 g/L (dw) was recorded (Fig. 2). Thus, the *Pseudochlorella pringsheimii* cannot grow without a nitrogen source and its biomass as a function of algae growth is directly proportional to the concentration of N in the medium. Regard to lipid content, the higher (28.14%) lipid content was got when grown in limited N of 0.125 g/ L N than that in rich or free nitrogen culture (Table 2). However, the N level in medium demonstrated significant negative correlations with the lipid contents in algae cells (*p* < 0.05). Thus, the *Pseudochlorella pringsheimii* can grow in N limited medium and accumulated lipid, with inverse relationship to the concentration of N in the medium. This result is in accordance with in same strain that the biomass was increased as function for increasing of N concentration [1, 4 and 5]. In general, nitrogen concentration significantly influences microalgae growth rate and their lipid content and lipid productivities, that depletion of N in cultivation medium causes a decrease in growth rate, but lipid productivities was increased. Therefore, a good relationship between the nitrogen dose and lipid content in the microalgae biomass was reported, which with nitrogen deficiency, the metabolic pathway of carbon fixation changes from protein synthesis to lipid production [21–24]. It can be concluded that nitrogen concentration favors higher biomass productivity and depletion of nitrogen shifts the flux to lipid production [7, 25–28].

Phosphorus is also essential element that plays an important role in microalgae growth, lipid production and fatty acid yield is required for metabolic processes such as energy transfer and signal transduction and photosynthesis (DNA, RNA and ATP) and nucleic acid synthesis [26]. As shown in Fig. 2, P concentrations in BBM medium (0 P free), (0.175 g/L optimum and double (0.350 g/L) had a high impact on growth and lipid content of *Pseudochlorella pringsheimii.* The increases of biomass dry weight was increased with increasing of P concentration and time, with highest correlation coefficient (*R*^2^ = ranged from 0.954 to 0.982). The biomass as a function for cells growth was slightly increased and in the absence of P free medium (0 g/ L free P) very slowly culture growth was observed. In contrast, in rich P sources (double the P, 0.350 g/L, rich P), the maximum biomass (dry weight dw) concentration of 1.356 g/L was recorded. The cultures grow in rich P medium and standard P BBM BG11 medium had highest biomass concentrations that in free P culture. The microalgae cultured in P-rich medium produced less lipid contents of 4.38% compares with that (12.36%) in P standard medium and in Free P (15.65%) medium. Thus, statistically, there is high significant difference between the lipid contents of P-rich or P- stander cultures and in the P-deficient BBM medium (*p* > 0.05). These results revealed that P is the most important nutrient for the cell proliferation and has significant impacts on cell growth of *Pseudochlorella pringsheimii* microalgae. The results were consistent with those previously reported in literature that the P deprived conditions led to increase the lipid content in *Phaeodactylum tricornutum, Chaetoceros sp., Isochrysis galbana* and *Pavlova lutheri* cultures [27–29]. In *Chlorella sp*. the higher lipid accumulation was observed with decreasing of P concentrations in nutrient media [30]. Fan et al. [31] who reported that the growth rate, biomass productivity and high accumulation lipids in *Meyerella* sp., *Chlamydomonas reinhardtii, Botryococcus* sp. and *C. pyrenoidosa* when cultured under nutrient stress condition. Abd El Baky and El Baroty [5] have also reported a decline in growth (dry weight) of microalgae under unfavorable conditions (P or N limitation) and is seems to be the most promising combination conditions for high lipid production.

The Fe ion as the most important trace metals has a significant effect in the growth rate, lipids and carbohydrates content in numerous microalgae. Ferric ions are involved in fundamental enzymatic reactions of photosynthesis and in regulating the gene expression and in metabolism pathway in algae [32]. As shown in Fig. 2, the biomass (dw) concentration in *Pseudochlorella pringsheimii* cells was increased gradually as Fe increased in nutrient medium. The biomass (dry weight dw) concentration were significantly inbreeded gradually by increases of Fe^3+^ iron concentration, with high correlation coefficient (*R*^*2*^ = 0.967 to 0.978). The similar trend was observed that the total lipid content was significant increased with increasing Fe ion (Table 2).The high total lipid content was found to be 19.25% were obtained in 10.0 mg/L Fe culture, which to be 2-folds) than that in free Fe^3+^ culture. Thus, in rich Fe medium, the microalgae biomass and its quality are closely related to microalgae growth rates, that a higher growth rate could produce a higher biomass and lipid content. This observation was corroborated was that reported by Abd El Baky et al. [5], who showed that Fe-rich conditions were suitable for lipid generation in *S. obliquus*. In microalgae *S. obliquus*, the total lipid content in cultures supplemented with high Fe^3+^ concentration exhibited a higher biomass dry weight than that in media supplemented with lower iron concentration. In *Botrycococcus spp*, the lipid content and lipid productivity were increased when grow with high iron concentration in combination with limited nitrogen condition [24]. Chew et al. [33] have demonstrated that *Monoraphidium* sp*.* algae can simultaneous increase of both lipid content and growth rate by increasing of bio-available iron Fe^3+^ concentration in the growth medium. It well know that that the increased concentration of iron in medium lead to an increased production of free radicals, which might have changed the metabolic pathway toward to lipids accumulation in the microalgae through increase the activity of acetyl-CoA carboxylase enzyme which, accelerating the biosynthesis of the fatty acids by carboxylation of acetyl-CoA to malonyl-CoA (precursors for lipid accumulation). Finally, high lipid content were obtained in either P starvation Nor in rich Fe ion cultures The relative levels of these effects on lipid content are similar to those on the growth as follows: N starvation > P starvation > Fe starvation. It interestingly, to known that the N and P starvation were the most effective strategy to increase the lipid content in microalgae cells.

The individual experimental showed that relative higher lipid content and lipid productivity was obtained in *Pseudochlorella pringsheimii* cultured under: N starvation, P starvation and rich Fe ions condition. Consequently, the microalgae was cultivated in photobioreactor (2000 L) in medium contained a combined (N, P and Fe) either nitrogen (1.25 g/L) and phosphorus (0.35 g/L) deficient and high concentration of iron ion (10 mg/L) and aerated with carbon dioxide (8% CO_2_) as a gases carbon source was selected as an effective strategy to production of biodiesel (Table 3 and Fig. 3).

Under indusial scales condition, the values of biomasses dry weight (4.0e5 g/L), lipid content (24.98%), lipid productivity (1.01 g/L) and total Chl a (96.31 mg/L) was calculated at late log phase (15 days). The specific growth rate μ (d^−1^), biomasses productivity biomass productivity (g^−1^ L ^−1^ d^−1^) was 0.274 and 0.270, respectively (Table 5). This result confirmed the fact that nitrogen and P starvation is the important nutrient for enhance of accumulation of lipid in microalgae. However, microalgae cultivated under optimal optimum conditions,, show high biomass growth but do not accumulate a large amount of reserve materials such as lipids which are useful for biofuel production [5, 7].

The direct transesterification TE reaction condition was optimized as a way to improve the biodiesel yield of algae lipids [3]. As shown in Table 4, The biomass of *Pseudochlorella pringsheimii* cultured at large scales in collective (N + Fe + P) medium subjected to a directly TE in one step to production of FAME biodiesel exhibited a high conversion percentage and biodiesel productivity was found to be about 97.64% and 0.067 mg^−1^L^−1^d^−1^, respectively (Table 4). These values are in specified limit (96.5%) reported for vegetable seed oils [34]. In many report that the Feedstock lipids (vegetable oils and microalgae lipid) were achieved maximum biodiesel yield ranged 70% to 97%, depending on the extraction-transesterification conditions [2–4, 35].

*Pseudochlorella pringsheimii* fatty acid FA) methyl esters profile was estimated by GC/MS analysis (Tables 5, 6). The data revealed that the most common fatty acid profiles consist mainly of C16:0, C18:0, C18:1 and C18:2 fatty acids (FAs). The fatty acid constituents have a high influence on fuel property [4, 5]. Since no individual fatty acid is responsible for any particular fuel property [36–38]. The composition percentage (%) of microalgae biodiesel were 33.98% saturated fatty acids (palmitic acid, stearic acid), 50.78% monounsaturated (C16 and C18 together) and 21.95% polyunsaturated fatty acids (C16 and C18 group together). A higher percentage of saturated fatty acids and monounsaturated could improve fuel property such as oxidative stability and cetane number of biodiesel of produced. Also, higher percentage of unsaturated and monounsaturated could be enhancing the biodiesel characteristic that is responsible for better cold flow properties and oxidative stability.

Table 7 shows the physical–chemical property of biodiesel of *Pseudochlorella pringsheimii* includes*:* the iodine value (IV), acid value (AV), saponification value (SV) and peroxide value (PV) that was determined based on ACOS method [14]. The results revealed that the low iodine value (IV, 89.84 ± 3.33 g I_2_/100 g lipid) is a good indicating parameter of the degree of saturation in fuel which has a high influences on the fuel viscosity and cold filter plugging point properties. The lower of AV (0.41 ± 0.09 mg KOH ^−1^ g) may be due to neutralized most of free fatty acids present in algae lipids during the trans-esterification process [3–5]. Also, the moderate saponification value (199.33 ± 1.66 mg KOH/g) indicates higher degree of lower molecular weight of the corresponding microalgae lipid and these results was similar that reported for olive (192) soybean (190)) and sesame (188 mg of KOH/g) crude oils that uses for production of commercial biodiesel. The CN value of *Pseudochlorella pringsheimii* was 53.46, which refluxed a good fuel property that the biodiesel quality is directly related to CN, an indicator of a fuel’s ignition quality in an engine and combustion quality [37]. In general, the value *Pseudochlorella pringsheimii* CN was within that values reported in *Desmodesmus abundan*s (54.89), *D. abundans* (58.36) and *D. obtusus*, (57.49) microalgae biodiesel, which could help ensure better cold start properties and minimize the formation of white smoke [38]. Also, similar results have been observed in other microalgae biodiesel of *Nannochloropsis sp., S. pectinatus* and *S. obtusus*. However, based on cetane hexadecane (C_16_H_34_) as a long straight-chain hydrocarbon (it is assigned of CN of 100) the most of the vegetable oil biodiesel has CN higher than 51 while the CN of petroleum base diesel (is a mixture of C 8–12 chain hydrocarbon) usually ranges from 40 to 52 [39].

*Pseudochlorella pringsheimii* biodiesel is characterized by low PV (< 0.10 ± 0.01 (O_2_meq/kg^1^ of lipid). is directly related to have a high oxidative stability of biodiesel against auto oxidation reaction. However, this value was expected to be low, due to no chance for generation of free radicals throughout the biodiesel preparation from algae lipids [2, 5]. Moreover, the low values of auto oxidative (OS, 0.169) and degree unsaturation (0.97) of microalgae biodiesel indicted that it has high auto-oxidation stability, which not need to add of any synthetic anti-oxidants (tert-butyl hydroquinone (TBHQ) or 3-tertbutyl-4-hydroxyanisole). On other hand, oxidation stability of biodiesel depends greatly on fatty acid compositions and ratio degree of unsaturated/saturated FAs, biodiesel contained high saturated FA is more stable than unsaturated ones [2]. Thus, *Chlorella* biodiesel exhibit superior oxidative stability due to presence of relatively high levels of saturated (SFA, %) and lower polyunsaturated fatty acids (PUFA).

The values of density (0.922 ± 0.080 at 15 °C, kgm^3^) and viscosity (4.98 ± 0.24 at 40 °C, mm^2^/s) were comparable with that recorded in the vegetable (D 0.85 – 0.95; KV 1.9–6.0 mm^2^/s) oils and it was dependent on the quantity of saturated FAMEs, which could be responsible for the moderate value of viscosity and density. However, the lower values of those parameters were desirable for improvements of the low temperature properties of biodiesel [37]. The heating value (39.22), LCSF (31.42) and CFPP (82.25), are used to evaluate biodiesel quality in terms of ignition readiness; combustion performance and fuel-line plugging temperature were within an acceptable range of EU biodiesel standards. According to those values the *Pseudochlorella pringsheimii* biodiesel tends to have a better ignition quality (CN values), and exhibit better flow performance at low temperatures (CFPP). These values were found to be within the limit values of international biodiesel fuel standards ASTM D 6751 [40] and EN 14,214 [41]. In general, the data of physical and fuel properties (density, CN, CEPP, acidity, and oxidative stability and heating value) of biodiesel from microalgae are comparable to those of conventional diesel. Overall, these results concluded that the *Pseudochlorella pringsheimii* is a suitable feedstock for biodiesel production due to its high oil content and related to their levels of saturated (palmitic C16:0) and mono-unsaturated lipids (oleic acids C18:1), which is advantageous for higher biodiesel quality.

## Conclusion

The microalgae oils represent a promising feedstock for biodiesel. Microalgae can be cultured to economical production of lipid through the nutrient stresses. The lipid content and biomass productivity as well as lipid composition of microalgae can be made nutrient stress. Thus, the optimization of the nutrient stress and cultivation process was applied to increasing the lipid content with provable fatty acid compositions for biodiesel production from *Pseudochlorella pringsheimii* microalgae. Under lab scales (2 L medium), the microalgae cultured in N- and P -limitation as well as Fe richest medium (in individual set), resulted a relative increase those parameters. The optimized value of (limited of N + P combined with high Fe) was choosing to cultivate of microalgae in large scale in the 2000 L PBR. Their collective of nutrients greatly improves the quantify lipid contents (25% w/w) and lipid productivity (74.07 mgL^−1^ day^−1^). The inducted lipid was converted to biodiesel via transestrification process yielded of 91.54 ± 1.43%. The fatty acid profile analyzes by GC/MS resulted in C16:0, C18:1, C18:2, C18:3 as a main constituents. Physical–chemical property (such as density, kinematic viscosity, gravity and cetane number) of the *Pseudochlorella pringsheimii* biodiesel was found to comply with ASTM D6751 and EN 14,214 standards, that thereby referring to high quality biodiesel.

## Data Availability

Not applicable.

## Abbreviations

PBR: Pseudochlorella pringsheimii
dw: Dry weigh
TL: Total lipid contents
AV: Acid value
SV: Saponification value
IV: Iodine value
PV: Peroxide value
M: Molecular weight
GC/MS: Gas chromatograph/ mass spectroscopy
ASTM: American Society for Testing and Materials
EN: European Committee for Standardization
